# In-hospital major arrhythmias, arrhythmic death and resuscitation after successful primary percutaneous intervention for acute transmural infarction: a retrospective single-centre cohort study

**DOI:** 10.1186/s12872-018-0851-z

**Published:** 2018-06-14

**Authors:** Marco Albanese, Korhan Alpaslan, Taoufik Ouarrak, Peter Merguet, Steffen Schneider, Wolfgang Schöls

**Affiliations:** 1 Herzzentrum Duisburg, Gerrickstr. 21, D-47137 Duisburg, Germany; 2 Stiftung Institut für Herzinfarktforschung, Bremserstraße 79 - Haus, MD-67063 Ludwigshafen a. Rh, Germany; 3 Klinik für Kardiologie und Angiologie, Herzzentrum Duisburg, Gerrickstr. 21, 47137 Duisburg, Germany; 4 Present address: Herzzentrum Hirslanden Zentralschweiz, Klinik St. Anna, St. Anna Str. 32, CH-6006 Luzern, Switzerland

**Keywords:** Myocardial infarction, Arrhythmias, cardiac, Ventricular fibrillation, Tachycardia, ventricular, Resuscitation, Death, sudden, cardiac

## Abstract

**Background:**

Transmural acute myocardial infarction (AMI) is associated with a high risk for ventricular arrhythmia before, during and after treatment. Consequently, it is recommended that patients diagnosed with transmural AMI be monitored in a cardiac care unit (CCU) so life-threatening arrhythmias can be treated promptly. We examined the incidence and timing of in-hospital malignant ventricular arrhythmias, sudden cardiac or arrhythmic death (SCD/AD) and resuscitation requirements in patients with transmural AMI recovering from percutaneous coronary intervention (PCI) undertaken within 12 h of symptom onset and without antecedent thrombolysis.

**Methods:**

This was a retrospective cohort study using the Duisburg Heart Center (Germany) cardiac patient registry. In total, 975 patients met the inclusion criteria. The composite endpoint was post-PCI ventricular fibrillation or tachycardia, SCD/AD or requirement for resuscitation. We compared the demographic and clinical characteristics of patients who met the composite endpoint with those who did not, recorded the timing of endpoint episodes, and used multivariable logistic regression analysis to identify factors associated with the endpoint criteria.

**Results:**

There was no significant difference in the length of CCU or hospital stay between the groups. In-hospital mortality was 6.5%, and the composite endpoint was met in 7.4% of cases. Malignant ventricular tachyarrhythmia occurred in 2.8% of the patients, and SCD/AD occurred in 0.3% of the cases. There was a biphasic temporal distribution of endpoint events; specifically, 76.7% occurred < 96 h after symptom onset, and 12.6% occurred 240–360 h after symptom onset. Multivariable regression analysis identified positive associations between an endpoint episode and the following: age (odds ratio [OR] 1.03, 95% confidence interval [CI] 1.01–1.05] per year); left ventricular ejection fraction (LVEF) < 30% (OR 3.66, 95% CI 1.91–6.99); peak serum creatine phosphokinase concentration (OR 1.01, 95% CI 1.00–1.02 per 100 U/dl); leucocytosis (OR 1.86, 95% CI 1.04–3.32), and coronary thrombus (OR 1.85, 95% CI 1.04–3.27).

**Conclusions:**

Most post-PCI malignant ventricular arrhythmias, SCD/AD and resuscitation episodes occurred within 96 h of transmural AMI (76.7%). A substantial minority (12.6%) of these events arose 240–360 h after symptom onset. Further study is needed to establish the influence of age, LVEF < 30%, peak serum creatine phosphokinase concentration, leucocytosis and coronary thrombus on post-PCI outcomes after transmural AMI.

## Background

Transmural acute myocardial infarction (AMI) is associated with a high risk for life-threatening arrhythmias in the early phase of an ischaemic event [1]. Current guidelines suggest that patients with transmural AMI be admitted to the intensive cardiac care unit (CCU) for 24–48 h after symptom onset [2]. Admission to the CCU allows prompt resuscitation in the event of cardiac arrest but has a substantial impact on treatment costs [3]. Although several models exist for predicting early complications in AMI, major arrhythmias have not been addressed in these models [4].

Mechanical reperfusion has become the treatment of choice for transmural AMI. Compared with thrombolysis, reperfusion has been shown to achieve higher rates of the best grade of coronary flow (Thrombolysis in Myocardial Infarction [TIMI] grade 3) and to reduce the incidence of recurrent ischaemia and reinfarction as well as infarct size, thus preserving left ventricular ejection fraction (LVEF) [5–7]. Nonetheless, there are limited data on the potential therapeutic benefit of primary percutaneous coronary intervention (PCI) in terms of the incidence of in-hospital malignant arrhythmias in patients with acute ST-elevation MI (STEMI). The incidence of in-hospital ventricular arrhythmias was not reported in large clinical trials, such as PRAGUE-2 or DANAMI-2 [7, 8]. A meta-analysis of large clinical trials indicates that the incidence of ventricular fibrillation (VF) appears to be lower in patients treated with PCI compared with that in patients treated with thrombolysis [1]. Several single-centre reports have yielded conflicting results on the incidence of in-hospital arrhythmias after primary PCI for acute STEMI. According to Giglioli and colleagues, no in-hospital life-threatening arrhythmias occurred after PCI; most life-threatening arrhythmias arose in the catheterization laboratory [9, 10]. Similarly, in a Swiss study, most arrhythmias occurred in the catheterization laboratory, nearly all within 24 h; a report from Germany revealed that 90% of fatal events occurred within the first 48 h [11, 12].

In our clinical practice, we were struck by the sudden cardiac death (SCD) of a 40-year-old patient 96 h after symptom onset in an otherwise apparently uncomplicated anterior STEMI. This patient satisfied none of the predictive factors, such as LVEF < 40%, commonly used for risk stratification. To better understand the timing of and risk factors for major arrhythmias in a current patient population treated by PCI, we retrospectively analysed the data of 975 patients treated at our tertiary care centre. Our analysis differed from that in the Swiss and German single-centre studies referred to above. As the analysis was limited to patients treated by primary PCI within 12 h of symptom onset and *without* antecedent thrombolysis, our cohort was a homogenous patient population that had only been exposed to the therapeutic consequences of PCI. In addition, we defined transmural AMI using angiographic evidence of an occluded vessel to ensure identification of transmural infarctions without ST-elevation on the surface electrocardiogram (ECG). A STEMI and this well-defined non-ST-elevation myocardial infarction (NSTEMI) subgroup were thus included in our cohort, as they represent a common pathoanatomical substrate associated with an increased risk of fatal events. We sought to establish whether 48 h of monitoring was sufficient to prevent fatal events and to determine whether clinical predictors of the occurrence of fatal events in our patient population differed from those described in previous publications.

Our primary objective was to determine the timing and total burden of in-hospital major arrhythmias after successful primary PCI for transmural AMI undertaken within 12 h of symptom onset without antecedent thrombolysis. The secondary objective was to identify clinical predictors associated with the occurrence of the combined endpoint of ventricular tachycardia (VT), SCD or arrhythmic death (SCD/AD) and resuscitation in the early phase of acute transmural infarction.

## Methods

### Study patients and data collection

**Fig. 1:**
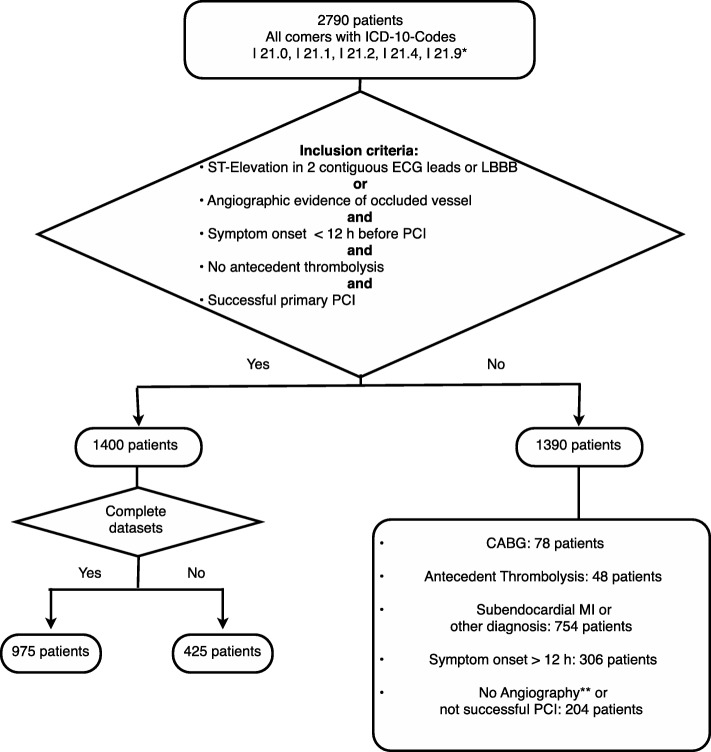
Study flow chart. * International Classification of Disease (ICD)-10 codes: I 21.0, acute transmural anterior myocardial infarction; I 21.1, acute transmural inferior myocardial infarction; I 21.2, acute transmural myocardial infarction of other sites; I 21.3, acute transmural myocardial infarction of unspecified site; I 21.4, acute subendocardial myocardial infarction; I 21.9, acute myocardial infarction, not precisely specified. ** no consent, death beforehand or contraindications. Other abbreviations: ECG, electrocardiogram; LBBB, left bundle branch block; PCI, percutaneous coronary intervention; CABG, coronary artery bypass grafting

This was a retrospective cohort study using our institution’s cardiac patient registry, into which data are input on discharge from hospital or after in-hospital death. Data collection began on January 1, 2005, and ended on May 17, 2011. We selected patients with symptom onset < 12 h before performance of PCI and evidence of transmural AMI as reflected by ST-segment elevation in at least two contiguous leads or the presence of left bundle branch block (LBBB) on the surface ECG or by angiographic evidence of AMI as determined by the presence of an occluded vessel. This definition of transmural AMI was chosen to ensure that transmural infarctions without ST-elevation were not missed; for example, left circumflex or right coronary artery occlusion without ST-elevation in the appropriate leads. Patients were excluded if they had undergone antecedent thrombolysis, if they were not treated by primary PCI, if primary PCI was not successful or if coronary artery bypass surgery was performed. Cardiogenic shock was not an exclusion criterion. The selection of patients in the cohort is shown in Fig. 1.

All patients were treated by a single group of cardiologists at the Duisburg Heart Centre, Duisburg, Germany, a tertiary care centre. Approximately 70% of the patients were admitted directly, with the remainder transferred from primary or secondary care centres where PCI was not available.

### Catheterization and percutaneous coronary intervention

Patients were treated with an oral loading of 300 mg clopidogrel. Further medical therapy was given, and the choice of bare metal or drug-eluting stents was made according to current national guidelines. Treatment with glycoprotein IIb/IIIa inhibitors or bivalirudin was performed at the cardiologist’s discretion. In most cases, only culprit lesions were treated by primary PCI, i.e., only the lesion of the infarct-related artery (IRA) was treated directly by angioplasty and stent delivery. Patients with cardiogenic shock instead underwent the most complete revascularization possible; specifically, all stenosed segments, including those vessels that were not the IRA, were treated by multiple PCIs during the acute intervention. Angioplasty success was defined as < 50% stenosis and a TIMI flow of grade 2 or 3; coronary thrombus was identified on angiography and by TIMI flow.

### Endpoints and definitions

The primary combined endpoints were the occurrence and timing of in-hospital ventricular arrhythmia, SCD/AD or resuscitation after primary PCI. All endpoints were either monitored or witnessed by experienced personnel or were ascertained by autopsy. Malignant ventricular arrhythmia or malignant tachyarrhythmia included VF or VT with haemodynamic compromise, and SCD was an unexpected death resulting from heart disease occurring within 1 h of symptom onset or unwitnessed overnight. Arrhythmic death was an unexpected death resulting from arrhythmia other than SCD or VT, i.e., complete heart block (CHB), pulseless electrical activity (PEA) or asystole. Resuscitation was defined as cardiocirculatory arrest followed by basic/advanced life support according to International Liaison Committee on Resuscitation (ILCOR) or American Heart Association (AHA) guidelines. Cardiac arrest was assessed by experienced medical and paramedic personnel based on telemetry detection of heart rhythm, blood pressure monitoring and pulse oxymetry or, in late events, on clinical evaluation. Any episodes before or during catheterization were not taken into account. Telephone follow-up was undertaken for the 7% of patients who had been transferred to other hospitals after primary PCI. No other follow-up was performed in this study, which focused on in-hospital events and the possible prevention of such events.

According to hospital policy, all patients were transferred to the CCU after PCI and were then continuously monitored for complex arrhythmia for a minimum of 48 h. Telemetry was used to monitor high-risk patients after transfer to the ward. Endpoints occurring throughout the entire hospital stay were analysed, and an autopsy was performed in unclear cases.

Normal left ventricular function was defined as an LVEF > 50%, slightly impaired function was defined as 40–50%, moderately impaired function was defined as 30–40% and severely impaired function was defined as < 30%. Contrast ventriculography or echocardiography was used to estimate LVEF immediately after admission.

### Statistical analysis

Absolute numbers and proportions (expressed as percentages) are used to describe the patient population. Medians (with quartiles) or means (with standard deviations) were calculated as appropriate. Categorical values were compared using the chi-square test, and continuous variables were compared using the two-tailed Wilcoxon rank sum test.

Multivariable logistic regression analysis was performed to identify independent predictors of the combined endpoint. The regression analysis took into account all events recorded. All baseline parameters previously reported to be associated with a higher risk for acute coronary syndrome were used as independent parameters in the model (Table 2) [4, 5]. *P* values< 0.05 were considered statistically significant. All *p* values were results of two-tailed tests. All analyses were performed using the SAS statistics programme (version 9.2; SAS Institute Inc., Cary, NC).

## Results

### Patient and treatment characteristics

The demographic and clinical characteristics of the patient population are shown in Table 1; the majority underwent PCI < 4 h after symptom onset (89.3%), and one or more stents were deployed in 98.7% of the cases (drug-eluting stents were deployed in 12.6% of the cases). Left ventricular function was severely impaired in 9.3% of the patient population. The mean duration of hospitalization was 10 d (range 7–35 d). Length of hospital stay was not associated with the occurrence of the combined endpoint. There was no statistically significant difference in the duration of CCU stay or of telemetric monitoring between patients who did or did not meet the composite endpoint.

**Table 1:** Baseline demographic and clinical characteristics of patients included in the study

	Total	Patients with composite endpoint	Patients without composite endpoint	*P*-value
Demographics				
Total, %	975	72 (7.4%)	903 (92.6%)	
Female, %	29.2% (285/975)	33.3% (24/72)	28.9% (261/903)	0.45
Age, (years)	62 (52–73)	68 (53–78)	62 (52–72)	< 0.05
Clinical presentation				
BMI, kg/m^2^	27 (25–30)	26 (25–29)	28 (25–30)	< 0.03
Adiposity (BMI > 30), %	24.0% (220/916)	12.7% (8/63)	24.9% (212/853)	< 0.05
Medical history				
Previous MI, %	14.0% (135/961)	24.3% (17/70)	13.2% (118/891)	< 0.05
Previous PCI, %	12.5% (120/960)	14.3% (10/70)	12.4% (110/890)	0.64
Previous CABG, %	3.3% (32/962)	7.1% (5/70)	3.0% (27/892)	0.06
Previous stroke, %	3.0% (29/963)	4.3% (3/70)	2.9% (26/893)	0.52
Hypertension, %	71.4% (688/964)	70.4% (50/71)	71.4% (638/893)	0.85
Hypercholesterolemia, %	74.9% (719/960)	59.7% (43/72)	76.1% (676/888)	< 0.01
Diabetes mellitus, %	24.0% (234/975)	29.1% (21/72)	23.5% (213/903)	0.27
Current smoker, %	46.9% (449/958)	31.9% (22/69)	48.0% (427/889)	< 0.01
PAD, %	5.7% (54/946)	13.0% (9/69)	5.1% (45/877)	< 0.01
Aspirin on admission, %	23.7% (221/957)	37.1% (26/70)	22.0% (195/887)	< 0.01
Laboratory on admission				
Potassium, mmol/L	4.0 (3.7–4.3)	4.2 (3.8–4.7)	4.0 (3.7–4.3)	< 0.05
Creatinine, mg/dl	0.9 (0.8–1.1)	1.1 (0.9–1.4)	0.9 (0.8–1.1)	< 0.0001
Elevated CRP (≥5×), %	10.3% (76/741)	14.3% (8/56)	9.9% (68/685)	0.30
WBC > 10.000 μl, %	57.0% (547/960)	72.0% (52/72)	55.7% (495/885)	< 0.01
HbA1c, %	8.4 ± 7.5	10.4 ± 13.8	8.1 ± 6.4	0.9
ECG on admission				
anterior STEMI, %	39.5% (383/970)	55.6% (40/72)	38.5% (343/891)	< 0.05
AF, %	4.4% (42/968)	10.3% (7/71)	3.9% (35/890)	< 0.01
LBBB, %	1.8% (17/965)	5.5% (4/72)	1.5% (13/888)	< 0.05
AV block, %	3.9% (38/968)	4.1% (3/72)	3.9% (35/820)	0.97
Coronary angiogram and intervention				
Three-vessel disease, %	27.3% (266/975)	31.9% (23/72)	26.9% (243/903)	0.36
Time to reperfusion < 4 h, %	89.3% (868/972)	92.4% (67/72)	89.6% (795/893)	0.35
Coronary thrombus, %	19.2% (187/973)	33.3% (24/72)	18.2% (163/894)	< 0.01
Stent implantation, %	98.7% (956/969)	97.2% (70/72)	99.5% (886/890)	< 0.05
GPIIa-IIIb antagonist, %	2.9% (28/965)	6.9% (5/72)	2.6% (23/887)	0.05
Bivalirudin, %	15.0% (145/968)	20.3% (15/72)	14.5% (130/889)	0.17
Clinical course				
CCU stay, h	46 (32–54)	49 (20–169)	46 (33–52)	0.17
Routine hospital ward stay, h	150 (120–193)	170 (105–200)	150 (120–193)	0.60
Telemetric monitoring, h	47 (33–66)	50 (13–192)	47 (33–58)	0.26
Use of vasopressors, %	12.5% (122/975)	83.5% (60/72)	6.9% (62/896)	< 0.0001
Maximum troponin value, U/L	3.4 (1.5–6.4)	5.9 (1.7–14.1)	3.3 (1.5–6.1)	< 0.01
Maximum CK value, mg/dl	1413 (549–2822)	2178 (609–4513)	1351 (545–2706)	< 0.01
Clinical outcome				
EF < 30%, %	9.3% (80/857)	43.4% (23/53)	7.1% (57/804)	< 0.0001
Death	6.5% (63/974)	72.2% (52/72)	1.2% (11/896)	< 0.0001
Cardiac death, % of total	88.9% (56/63)	96.2% (50/52)	54.5% (6/11)	< 0.001

Troponin was assayed as troponin T (normal < 0.01 U/L)

*Abbreviations*: *BMI* body mass index, *MI* myocardial infarction, *PCI* percutaneous coronary intervention, *CABG* coronary artery bypass grafting, *PAD* peripheral artery disease, *CRP* C-reactive protein, *WBC* white blood cell count, *Hba1c* glycated hemoglobin, *STEMI* ST-elevation MI, *AF* atrial fibrillation, *LBBB* left bundle branch block, *AV* atrioventricular, *GP* glycoprotein, *CCU* coronary care unit, *CK* creatine phosphokinase (normal < 250 mg/dl), *EF* ejection fraction

The combined endpoint was observed in 7.4% of patients. PEA and VT were the most common malignant arrhythmias. Resuscitation was undertaken in 7.1% of the patients. By analysing the patient population according to the presence or absence of the combined endpoint, we observed the following. Patients who met the endpoint criteria were older, more frequently had a history of previous MI and peripheral arterial disease, more often took aspirin, and were more frequently diagnosed with anterior STEMI, atrial fibrillation or LBBB on admission. Serum troponin and creatine phosphokinase (CK) concentrations were almost doubled, and a higher proportion had an elevated white blood cell count (WBC) compared with those who did not meet the composite endpoint.

During coronary intervention, patients who subsequently met the composite endpoints were found to have a more extensive coronary thrombus, and there was a tendency towards more frequent use of glycoprotein IIb/IIIa inhibitors. More patients with severely impaired LVEF (< 30%) or who required vasopressor support subsequently met the combined endpoint. There was no difference in coronary status or time to reperfusion between the two groups. Although the difference in stent deployment rate was statistically significant (*P* <  0.05), it was not considered relevant because of the small difference in absolute numbers (97.2 vs 99.5%).

Descriptive analysis of the data showed that 27.5% of the patients who met the endpoint underwent early perfusion (< 90 min), 64.9% underwent intermediate perfusion (1.5–4 h), and 7.6% underwent late reperfusion (> 4 h). There was no significant difference in the proportions who developed VT (21.6, 18.6 and 18.6%, respectively) or AD (32.5, 32.2 and 41.9%, respectively) or who required resuscitation (45.9, 49.2 and 41.9%, respectively).

### Predictors of the composite endpoint

Of the 12 original candidate variables, five remained statistically significant in the multivariate analysis and formed a final set of predictor variables (Table 2). These variables were age, WBC > 10,000/l, coronary thrombus, LVEF < 30% and peak serum CK concentration. The presence of atrial fibrillation was not a significant predictor (*P* = 0.051).

**Table 2:** Predictors of the composite endpoint in patients with acute myocardial infarction (multivariate analysis)

	ß Coefficient	*P* value	Odds ratio (95% CI)
Female	0.3059	0.6699	0.878 (0.48–1.59)
Age, years	0.0118	0.008	1.032 (1.008–1.05)
PAD	0.4613	0.4581	1.408 (0.57–3.47)
anterior STEMI, %	0.2774	0.4000	1.263 (0.73–2.17)
AF, %	0.4641	0.0514	2.470 (0.99–6.13)
LBBB, %	0.7615	0.3608	2.006 (0.45–8.92)
Coronary thrombus, %	0.2909	0.0339	1.853 (1.04–3.27)
EF < 30%	0.3307	< 0.0001	3.656 (1.91–6.99)
Creatinine, mg/dl	0.0037	0.9077	1.000 (0.99–1.00)
WBC > 10.000 μl	0.0118	0.0354	1.863 (1.04–3.32)
Maximum troponin value, U/L	0.0009	0.7892	1.000 (0.99–1.00)
Maximum CK value, 100 U/dl	0.0032	< 0.0001	1.014 (1.00–1.02)

*Abbreviations*: *CI* confidence intervals, *PAD* peripheral artery disease, *STEMI* ST-elevation myocardial infarction, *AF* atrial fibrillation, *LBBB* left bundle brunch block, *EF* ejection fraction, *WBC* white blood cell count, *CK* creatine phosphokinase

### Temporal distribution of endpoints and correlation with endpoint predictors

**Fig. 2:**
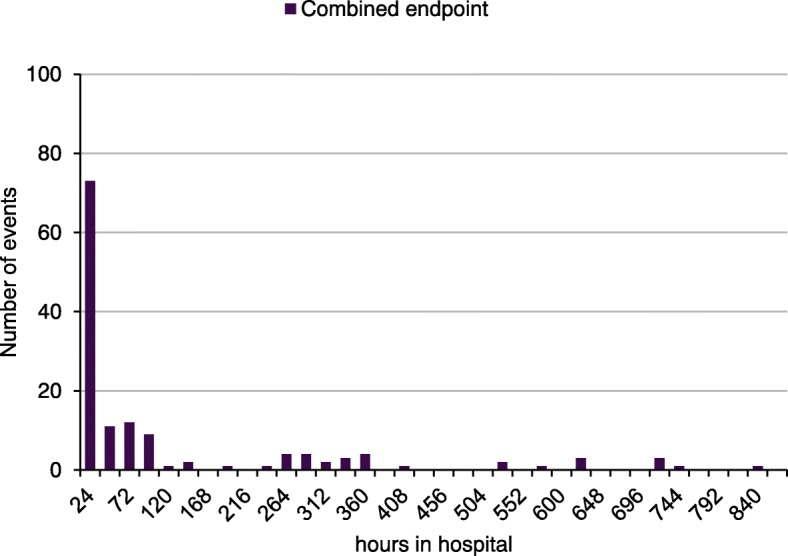
Distribution of time of events of the combined endpoint in transmural AMI after reperfusion by primary PCI without antecedent thrombolysis. The combined endpoint comprises VT, resuscitation and SCD/arrhythmic death. Multiple events per patient were possible: 24 h: 73; 48 h: 16; 72 h: 11; 96 h: 9; 120 h: 1; 144 h: 2; 168 h: 0; 192 h: 1; 216 h: 0; 240 h: 1; 264 h: 4; 288 h: 4; 312 h: 2; 336 h: 3; 360 h: 4; 384 h: 0; 408 h: 1; 432 h: 0; 456 h: 0; 480 h: 0; 504 h: 0; 528 h: 1; 552 h: 0; 576 h: 1; 600 h: 0; 624 h: 3; 648 h: 0; 672 h: 0; 696 h: 0; 720 h: 3; 744 h: 1; 768 h: 0; 792 h: 0; 816 h: 0; 840 h: 1

We observed a biphasic distribution of events: a total of 62.7% of the combined endpoints occurred within the first 48 h of symptom onset, and a total of 76.7% were recorded within 96 h of symptom onset. Between 240 and 360 h following symptom onset, the incidence of the combined endpoint was 12.0%. A smaller number of endpoint events occurred later in the clinical course (Fig. 2).

Specifically, 12.7% of malignant ventricular arrhythmias and 21.6% of resuscitations occurred within the first 48 h of symptom onset. In addition, 13.4% of VT/VF and 31.7% of resuscitations were recorded within 96 h of symptom onset. Between 240 and 360 h following symptom onset, the incidence of ventricular arrhythmia was 1.4% and that of resuscitation was 4.2%. In the first 48 h after AMI, 25.4% of the SCD/AD episodes were observed, and a total of 31.7% occurred within 96 h of symptom onset. The incidence of SCD/AD between 240 and 360 h following symptom onset was 7%. The exact timing of events is shown in Table 3 (*supplemental data).*

**Table 3:** Occurrence of the specific components of the combined endpoint in time

Adverse event	24 h	48h	72 h	96 h	120 h	144 h	168 h	192 h	216 h	240 h
VT/VF	18		1							
SCD/AD	25	11	5	4	1	1				1
Resuscitation	30	5	5	5		1		1		
Adverse event	264 h	288 h	312 h	336 h	360h	384 h	408h	432h	456h	480h
VT/VF		1		1			1			
SCD/AD	3	1	2		3					
Resuscitation	1	2		2	1					
Adverse event	504 h	528 h	552 h	576 h	600h	624h	648h	672h	696h	720h
VT/VF						1				1
SCD/AD		1		1		1				
Resuscitation						1				2
Adverse event	744h	768h	792h	816 h	840h					
VT/VF										
SCD/AD	1				1					
Resuscitation										

Depicted are absolute numbers and multiple events per patient were possible. A total of 142 events divided into specfic events as follows: a) Ventricular tachycardia (VT) 24 events b) SCD/arrhythmic death 62 events c) Resuscitation 56 events

Due to the low number of events, it was not possible to calculate a risk score. Therefore, we analysed the data for the presence of predictor variables in patients with endpoints that occurred > 48 h after admission. In 90.0% of the patients with endpoints > 48 h after admission, a minimum of two or more predictors was present (data not shown).

### Secondary outcomes

Total in-hospital mortality was 6.5%. Most of the deaths were from a cardiac cause (88.9%) and 77.0% were due to arrhythmia. A minority of cardiac deaths (8.7%) occurred unobserved late in the clinical course (> 96 h after admission). Patients experiencing sustained VT or VF had in-hospital mortality rates of 15.4 and 93.3%, respectively. Only 0.8% developed CHB after successful PCI. Among the patients who developed PEA, VF, CHB or asystole, cardiogenic shock was present in 86.7% of patients with PEA and 53.3% of patients with VF (Table 4).

**Table 4:** In-hospital arrhythmias: relationship to cardiogenic shock and relative mortality

Malignant arrhythmia	% of total^a^	Cardiogenic shock^b^	Mortality^b^
Tachyarrhythmia			
sustained VT, %	1.3%	15.4%	15.4%
VF, %	1.5%	53.3%	93.3%
Bradyarrhythmia			
CHB, %	0.8%	37.5%	62.5%
Bradyarrhythmia absoluta, %	0.2%	0%	0%
Sinus arrest,	0.1%	0%	0%
SCD, %	0.3%	0%	100%
PEA, %	2.6%	86.7%	100%
Asystole, %	0.8%	53.8%	84.6%

*VT* ventricular tachycardia, *VF* ventricular fibrillation, *CHB* complete heart block, *SCD* sudden cardiac death, *PEA* pulseless electrical activity

^a^ with respect to entire study population

^b^ with respect to cases of VT, VF, CHB, Bradyarrhythmia absoluta, sinus arrest, SCD, PEA, Asystole

## Discussion

### Key findings

The primary objective of this study was to determine the total burden and timing of in-hospital major arrhythmias and of the combined endpoint of ventricular arrhythmia, SCD/AD or resuscitation after successful primary PCI for transmural AMI within 12 h of symptom onset. Malignant ventricular tachyarrhythmia occurred in 2.8% of the patients, bradyarrhythmia occurred in 1.1%, PEA occurred in 2.6%, asystole occurred in 0.8% and SCD occurred in 0.3%. The combined endpoint was met in 7.4% of patients. We observed a biphasic distribution of events, with 76.7% of endpoints occurring within 96 h of symptom onset and 12.6% occurring 240–360 d after AMI. Occasional events were, however, observed throughout the hospital stay.

The secondary objective was to identify clinical predictors associated with the occurrence of the combined endpoint, ventricular arrhythmia, SCD/AD or resuscitation in the early phase of acute transmural infarction. Due to the low number of events, we were unable to reliably identify clinical predictors but observed positive associations between the combined endpoint and age, severely impaired LVEF, peak serum CK concentration, leucocytosis and coronary thrombus in the multivariable regression analysis.

### Comparison with other studies

It is difficult to compare our findings with other studies on PCI, which were designed differently and had different endpoints. In some studies, more time elapsed between symptom onset and PCI than in our cohort. In other studies, the occurrence of endpoints before and during cardiac catheterization were taken into account, and the length of observation time and the differentiation of arrhythmias were not the same in all studies. The studies were, however, broadly comparable regarding study populations, concomitant medications, techniques and stents used.

The incidences of sustained VT or VF and SCD in our registry were 2.8 and 0.3%, respectively. The total burden of in-hospital malignant ventricular arrhythmias in our registry was therefore similar to or lower than that described in other reports of transmural AMI treated by primary PCI. In a similarly designed study by Giglioli et al., only episodes of VF were recorded, which occurred in 0.6% of patients after cardiac catheterization; however, the absence of reports on other endpoints makes a direct comparison with their findings difficult. In our study, the time to reperfusion was less than 4 h after symptom onset in 89.3% of the patients, and the results are probably best compared with the following two studies, which both included patients with STEMI treated with primary PCI within 6 h of symptom onset. Mehta et al. reported in the APEX-AMI trial that 2.0% of patients developed VT or VF after cardiac catheterization [13]. Furthermore, Mehta et al. undertook an analysis of outcomes from the HORIZONS-AMI trial and reported that 5.2% of patients developed VT/VF after PCI [14]. Only a limited comparison is possible with the following studies because the time to primary PCI after symptom onset was longer in those studies. An analysis by Ohlow of an observational registry of patients with STEMI treated with primary PCI within 24 h of symptom onset revealed that the incidence of malignant arrhythmia was 4.7%; however, the investigators did not state where the arrhythmias occurred, and they observed endpoints only during the CCU stay [12]. A single-centre retrospective cohort study of patients with STEMI treated with primary PCI within 24 h of symptom onset undertaken by Cricri and colleagues reported a comparable number of patients (2.6%) who developed VT or VF after cardiac catheterization [11].

There are limited data on the potential therapeutic benefit of primary PCI compared to thrombolysis in terms of the incidence of in-hospital malignant arrhythmias in patients with acute STEMI. The incidence of malignant ventricular arrhythmia in our cohort was less than the VF or sustained VT incidence of 10.2% reported in the GUSTO-I study, a large randomized clinical trial investigating thrombolysis with streptokinase in patients with STEMI within 6 h of symptom onset [15]. This observation corroborates the hypothesis of PCI being superior to thrombolysis.

We observed a predominantly biphasic distribution of composite endpoint events, with 76.7% occurring within 96 h of symptom onset and 12.6% occurring between 240 and 360 h. This biphasic pattern differs from the more monophasic distribution observed in the thrombolysis era as well as in studies of major arrhythmias after successful primary angioplasty for acute STEMI. In the GUSTO-1 thrombolysis trial, 39 and 55% of in-hospital deaths occurred within 24 and 48 h of randomization, respectively, while 84% of malignant arrhythmias occurred within 48 h of randomization [16]. In study settings similar to ours with patients treated within 6 h of symptom onset, Mehta and colleagues found, in retrospective analyses of the APEX-AMI study population and in the prospective HORIZONS-AMI trial, that 70 and 85%, respectively, of VT-associated fatal events occurred within the first 48 h of leaving the catheterization laboratory [13, 14]. In the two studies that included patients with STEMI treated with primary PCI within 24 h of symptom onset, a different temporal distribution was observed. In the study of Cricri and colleagues, most of the malignant arrhythmias (sustained VT, VF or bradycardia necessitating cardiac pacing) developed in the catheterization laboratory, and nearly all of these arrhythmias occurred within 24 h [11]. Ohlow and colleagues reported 90% of VTs occurring within the first 48 h [12].

Our secondary objective was to identify clinical predictors associated with the occurrence of the combined endpoint of VT, SCD or arrhythmic death, and resuscitation in the early phase of acute transmural infarction. These predictors would a) identify patients at high risk for the combined endpoint at the time of hospitalization and b) identify patients at risk despite the apparent lack of established risk factors, e.g., cardiogenic shock.

The variables used for our logistic regression modelling were based on observations from prior studies of risk stratification and include patient demographic and clinical characteristics, measures of the acuity and angiographic presentation of the MI, and indicators of the type and extent of myocardial ischaemia and necrosis [4, 5]. Our results indicate that age, severely impaired LVEF, peak serum CK concentration, leucocytosis and the presence of coronary thrombus were positively associated with the combined endpoint. In patients treated by primary PCI in the APEX-AMI trial as well as those in the study of Ohlow and colleagues, a post-procedural TIMI flow of less than grade 3 was associated with VT or VF [12, 13]. In the APEX-AMI trial, leucocytosis was also a predictor of ventricular arrhythmia [13]. A similar observation was also made by Rahimi et al. in patients with NSTEMI [17].

In several studies from the thrombolysis era, age, severely impaired LVEF and peak serum CK concentration have also been consistently associated with a higher incidence of VF or VT during or immediately after AMI. An analysis of the Holter Registry data from the Cardiac Arrhythmia Suppression Trials showed that age and reduced LVEF were independent predictors of the incidence and frequency of VT [18]. The analysis by Ruiz-Bailén and colleagues of the ARIAM Database also showed that age and peak CK concentration were associated with VF [19]*.* In the study by Mont and colleagues of patients with AMI who were referred to a CCU after thrombolysis, serum CK-MB fraction concentration, Killip class and bifascicular block were independent predictors of the development of sustained monomorphic tachycardia [20].

We also reported on secondary outcomes and found that in our ‘real-world’ single-centre registry of patients with acute transmural infarction treated with primary PCI, total in-hospital mortality was 6.5%, which is consistent with other reports. In similarly designed retrospective single-centre studies by Giglioli et al. and Kozieradzka et al., in-hospital mortality was 5.9% and 30-d mortality was 6.3% [4, 10]. A more recent small single-centre study in China showed that mortality was 8.6% in patients aged > 60 years compared with 1.5% in the non-elderly group [21]. In our cohort, 15% of patients with sustained VT and 93% of patients with VF died, resulting in a mortality rate among those who developed a ventricular arrhythmia twice that in a retrospective cohort study of 2317 patients with AMI reported by Henkel et al. (mortality rate 38%) and the APEX-AMI study (mortality rate 33%) [1, 13]. The incidence of bradyarrhythmia in our cohort was also lower than that in other reports; specifically, only 1.3% of the patients developed bradyarrhythmia (with 0.8% of the cases being CHB), while Giglioli et al. reported an incidence of 6.3% [10].

### Possible mechanisms and explanations

Our observation of a biphasic temporal distribution of the combined endpoint can be explained by the nature of our chosen endpoint, which comprised episodes of all major ventricular arrhythmias, SCD/AD and resuscitation not only during the initial phase when patients were continuously monitored in the CCU but also during the entire hospital stay. In addition, we did not consider any events that occurred before or during cardiac catheterization [9, 11]. Other potential explanations are that the time to PCI was longer (up to 24 h) in other studies [11, 12], and thus, myocardial necrosis may have been more pronounced in these studies. Furthermore, our population was unselected, unlike trials of study drugs/drug-eluting stents, such as the APEX-AMI and HORIZONS-AMI trials, in which some potential participants were excluded [13, 14].

The multivariate analysis identified variables associated with the composite endpoint that differed from those of other studies, potentially because our endpoint included all ventricular arrhythmias, SCD/AD and resuscitation episodes, while other studies used only ventricular arrhythmias and CHB as the endpoint. Furthermore, leucocytosis, elevated CK concentration and severely impaired LVEF would not have been a consequence of staged or advanced infarctions in our cohort, as we only included patients with < 12 h of symptoms in whom necrosis and reactive inflammation would not have become established. In addition, 92.4% of our patients underwent primary PCI within 4 h of symptom onset.

It is challenging to explain the relatively high mortality rate of those who developed arrhythmia in our cohort. We cannot conclude that adverse outcomes were due to cardiogenic shock and VF alone, as > 40% of the patients in our cohort who died after an episode of VF did not exhibit symptoms or signs of low cardiac output.

Barron et al. concluded in the retrospective analyses of a TIMI 10 thrombolysis study that an elevated WBC was associated with reduced epicardial blood flow and myocardial perfusion, thromboresistance (arteries open later and have a greater thrombus burden), and a higher incidence of new congestive heart failure and death [22]. Our data therefore seem to suggest that inflammation and the WBC itself may also be directly correlated with coronary thrombosis, impaired perfusion, and reperfusion injury in the PCI era.

### Study strengths and limitations

Our cohort comprised 975 multi-ethnic patients with a clearly defined pathophysiological substrate (transmural AMI) and therapy (only primary PCI for reperfusion of the occluded vessel). In this cohort, we found a lower incidence of in-hospital major arrhythmia, SCD/AD and resuscitation but a higher mortality rate and biphasic temporal distribution of those who met the composite endpoint. Multivariable regression analysis showed positive associations between several factors and the combined endpoint. Due to the low number of events, we were unable to develop and calculate a risk score for the occurrence of the combined endpoint. The low number of events may be a consequence of the retrospective nature of this study.

A major concern may relate to the period in which data were collected (2005–2011) and the procedural aspects, i.e., the P2Y12 inhibition provided (Clopidogrel) and the very low percentage of implanted DES during primary PCI (12.6%) in our study. In the EUROMAX trial, the choice of prasugrel or ticagrelor over clopidogrel was not associated with differences in acute stent thrombosis or 30-day ischaemic outcomes after PCI [23]. Furthermore, in the PRAGUE-18 study, prasugrel and ticagrelor were found to be similarly effective during the first year after MI, and economically motivated early post-discharge switches to clopidogrel were not associated with an increased risk of ischaemic events [24]. The low percentage of implanted DES (all first generation) in our study should not be of concern because a meta-analysis [25], cost analysis data [26], cohort registries [27] as well as single-centre studies [28] have shown that the only benefit of DES is the reduction of target vessel revascularization. Total mortality and MACE or stent thrombosis are not superior in patients with STEMI receiving bare metal stents (BMS), although a trend toward lower mortality may be seen with everolimus-eluting stents (EES) [29]. Furthermore, in the Norwegian Coronary Stent (NORSTENT) trial, patients (26% with STEMI) were randomized to the DES or BMS group. There were no differences in the incidence of the primary endpoint (a composite of death from any cause or non-fatal spontaneous MI) after a median follow-up of 5 years [30].

Another limitation is the potential lack of generalizability of this single-centre study, which may not reflect outcomes achieved by other teams in different settings. Furthermore, observational studies may generate only a hypothesis that remains to be proven in a randomized controlled trial [31].

## Conclusions

In this population of patients with acute transmural myocardial infarction treated with early PCI, we found that the total burden of malignant ventricular arrhythmias was reduced but that the mortality rate of those who developed these arrhythmias was substantially higher. Reperfusion by PCI resulted in a biphasic distribution of all major ventricular arrhythmias, SCD/AD or resuscitation. This result differed from the single peak pattern described in previous studies with thrombolysis as well as with primary PCI.

Nearly one-third of malignant arrhythmias and/or deaths occur late in the clinical course, and predictors are necessary to prevent such events. Due to the low numbers of events and the retrospective nature of this study, more evidence of the predictive value of the identified variables is needed before firm conclusions can be drawn. At this point, we would not recommend any change in clinical practice, as there is no basis for an algorithm to identify patients at risk. Our findings should, however, prompt a multi-centre study to examine the predictors that we have identified in greater detail, to establish a risk scoring system, and to investigate the influence of preventive measures, such as longer periods of monitoring and the use of wearable defibrillator vests.

## Abbreviations

AD: Arrhythmic death
AMI: Acute myocardial infarction
BMS: Bare metal stent
CCU: Coronary care unit
CHB: Complete heart block
CK: Creatine phosphokinase
DES: Drug-eluting stent
ECG: Electrocardiogram
EES: Everolimus-eluting stent
EF: Ejection fraction
ICU: Intensive care unit
IRA: Infarct-related artery
LAD: Left anterior descending
LBBB: Left bundle branch block
LV: Left ventricular
LVEF: Left ventricular ejection fraction
M: Myocardial infarction
NSTEMI: Non-ST-elevation myocardial infarction
PAD: Peripheral artery disease
PCI: Percutaneous coronary interventions
PEA: Pulseless electric activity
SCD: Sudden cardiac death
STEMI: ST-elevation myocardial infarction
TIMI: Thrombolysis in myocardial infarction
VF: Ventricular fibrillation
VT: Ventricular tachycardia
WBC: White blood cell count
